# Virginity Control and Hymen (re)Construction: Gender Analysis from the Perspective of Young Women

**DOI:** 10.1007/s10508-025-03272-6

**Published:** 2025-10-31

**Authors:** Monica Christianson, Carola Eriksson

**Affiliations:** Department of Nursing, Umeå University, 901 85 Umeå, Sweden

**Keywords:** Gender perspective, Grounded theory, Sexuality, Virginity, Women, Hymenoplasty

## Abstract

Virginity is a social construct with no medical or scientific value. Although some people believe an intact hymen is proof of virginity, this belief has not been confirmed using forensic evidence. Despite these facts, in many places in the world women’s sexuality is controlled via virginity testing and hymen (re)constructions. These practices are on the rise globally, including in Sweden. Voicing the viewpoints of racialised women are rare. Using a gender perspective, this study analysed how young women living in Sweden who experience patriarchal chastity norms construct and understand virginity and what conditions, actions, and consequences follow when virginity is highly valued. A total of 14 young women originating from countries in the Middle East, East Africa, and Sweden were interviewed. This study uses constructive grounded theory to explore concepts such as oppression, inequality, and injustice. The category Unequal sexual conditions for women compared with men describes why virginity is understood as a troublesome condition for women. The category The making and faking of a virgin presents various ways women’s sexuality is controlled in cultural and medical contexts. The consequences of the intrusions of medicine and the roles physicians play are included in the category Surgical interventions in gendered bodies. The emergent core category, Intersecting dimensions of honor cultures, (un)medical power, and gender injustice sustain the norms of virginity, explains how the epistemologies of ignorance are connected to virginity and the hymen. Unscientific discourses about virginity testing and the hymen (re)construction must be challenged if we intend to stop these harmful practices.

## Introduction

Virginity, a social construct, has no scientific basis, and the state of virginity cannot be verified through a genital examination (Crosby et al., 2020). As virginity cannot be seen, it cannot be measured. According to the WHO (2018), the concept of virginity is a social, cultural, and religious construct with neither a medical nor a scientific basis. Despite these facts, virginity testing and hymen (re)constructions appear to be global phenomena that are on the rise in the Middle East (Wynn & Hassanein, 2017) and in the Western world (Crosby et al., 2020; Konaç, 2024; Moaddab et al., 2016; Moussaoui et al., 2022). As such both virginity testing and hymen (re)construction reflect patriarchal norms that create intimidating environments for women (Wild et al., 2015). Here, we ground our analysis in the voices of young women who have experienced honor cultures (e.g., a social system where an individual’s reputation and social standing are central) and have knowledge about the socially constructed meanings of virginity.

Virginity has long been claimed as one of the globally circulated gendered myths that affect women’s sexual health in many ways (Christianson & Eriksson, 2013, 2014, 2015, 2021; Militz, 2022). Historically, virginity has been a marker of purity as it is used to control women’s sexuality (Sissa, 2013). For western feminists, the creation of the concept is both a consequence and a result of living in patriarchal societies, where men’s control of women’s sexuality is considered an essential part of culture (Simga, 2019 p. 113). According to Simga, women in these societies are discriminated against to a higher extent than men—i.e., sexual discrimination and control are more often experienced by women than men. The control of women’s sexuality is discussed in an article from Africa where the data comprised of messages and posts on Facebook show that virginity could pave the way for a happy marriage or protect young people from sexually transmitted infections (Olamijuwon & Odimegwu, 2022).

### Virginity Testing

Virginity testing may be done by female relatives within the home or in the community (WHO, 2018). For some, virginity is demonstrated on the wedding night rather than in advance of the wedding whereas for others it is done in advance of a wedding or is noted as part of routine gynecological care. The purpose of virginity testing, done during a gynaecological check-up, is to evaluate whether a woman has engaged in vaginal intercourse (Independent Forensic Expert Group, 2015), a practice that assumes girls are born with a thin membrane in the vagina that ruptures on first vaginal intercourse and results in bleeding. However, according to a systematic review of virginity testing, which includes 17 studies relying on medical evidence, it is not possible to determine who has and who has not engaged in vaginal intercourse using this criteria (Olson & García-Moreno, 2017). Despite the fact that a woman’s virginity is constituted more in social discourse and imagination than in any biological reality (Christianson & Eriksson, 2011; Delgross, 2019; Wynn & Hassanein, 2017; Zayed & Saffaa, 2022), virginity testing is performed in many parts of the world, most commonly in Asia, the Middle East, and countries in northern and southern Africa. Due to increased globalisation, requests for and cases of virginity testing are also reported in Canada, North America, Belgium, Spain, and Sweden, countries that have no known widespread history of this phenomenon (WHO, 2018).

According to WHO (2018), virginity testing is referred to as two-finger testing, genital inspection, or vaginal examination. The WHO states that virginity testing is a violation of human rights, rooted in discrimination of women and girls. Both short- and long-term consequences result from such testing, including physical, mental, and sexual health problems (Independent Forensic Expert Group, 2015; WHO, 2018). In a Turkish context, where honor (*namus*) often is valued, the connotations are very different for women and men (Şimga, 2019). Women experience strict control over their chastity, are forced into marriages, often at a young age, and denied sexual and reproductive rights, often resulting in depression that sometimes leads to suicide; moreover, unwed women who are discovered not to be “virgins” can even be murdered by a male relative (so-called honor killings) (Şimga, 2019). AlQahtani et al., (2023), in a narrative review, revealed how important the sociocultural gender norm of being the “man in the house” can be. Men who live up to the expectation of enforcing control over their wives or female relatives gain respect, and men who do not maintain control over their wives or female relatives, especially when it comes to “protecting” their virginity, lose respect, reflecting a fragile masculinity and risk of ostracization from the community.

### Hymen (re)Construction

Although genital variability differs, the presence of a hymen has been claimed to confirm a woman’s virginity, but in contemporary societies no forensic evidence has been found that proves the presence of a hymen indicates virginity (Delgross, 2019; Independent Forensic Expert Group, 2015; Kotb & Abo-Zeid, 2022). The hymen is an embryological remnant of mesodermal tissue that usually perforates during the later stages of embryo development and does not exist after birth (Dane et al., 2007) In medical terminology, hymen (the term hymen comes from the Greek god of marriage and weddings, “Hymenaeus”), is a controversial “non-existing part” in women’s genitals (Hobday & Dayton, 1997). However, across cultures and times the mythologisation and fetishization of the hymen has influenced young women’s sexual health in many ways (Delgross, 2019), exposing young women to unscientific practices such as virginity testing and hymen (re)construction (Christianson & Eriksson, 2014; Konaç, 2024, Kotb & Abo-Zeid, 2022; WHO, 2018). At least one narrative literature overview has discussed hymen (re)construction in Middle East and North Africa countries (MENA) and in Europe (Saharso, 2022). According to Saharso (2022), hymen (re)construction is a controversial issue, viewed as either undermining women’s morale or strengthening women’s autonomy.

Evidence suggests that vaginoplasty without medical indications, including hymen (re)construction, is increasing globally (The Aesthetic Society (2020–2021). In a literature review of 82 articles, hymen (re)construction is labelled as “renewal of virginity”, hymenoplasty, or hymenorrhaphy, and three surgical methods are described in detail: the approximation method, the cerclage method, and the suture three stratums around the introitus (STSI) method (Wisniewska-Slepaczuk, et al., 2022). These outpatient methods require local or general anaesthesia and include absorbable sutures in the vagina or, as in the STSI method, the removal of vaginal tissues and three layers of stitches, which increases the likelihood of bleeding during sexual intercourse. Surprisingly, the methods do not leave any scars, but trained gynaecologists can often identify whether a patient has received such treatments. Although many believe that these methods do not result in many complications (Konaç, 2024), a Belgian study that interviewed gynaecologists found that bleeding, minor infections, hematoma, or dyspareunia can occur as the result of these methods (Leye et al., 2018). As most data on hymen (re)constructions are lacking, these operations are not recommended for women under the age of 18, and physicians are advised to safeguard that young women are not coerced into having the surgery. Therefore, physicians should not promote or advertise the procedure (Vieira-Baptista et al., 2018).

Hymen (re)constructions are performed within Swedish public healthcare, but the breadth of the phenomenon is unclear as a diagnosis that requires such an operation and the operation itself lack a precise description (Christianson & Eriksson, 2004). In the records, physicians tend to choose wordings such as “other diagnosis” or “vaginal plastic surgery.” Although virginity testing has no scientific value, it is practiced in (and outside) medical settings in Sweden. In 2015, a Swedish television program revealed that so-called virginity certificates were issued at Swedish healthcare centres (Swedish doctors ‘give teenage girls virginity tests’ against their will’; Kalla Fakta [2015]). Recently, the Swedish government has intensified investigations about honor-related violence and oppression and has initiated an ongoing investigation concerning how to criminalize virginity testing, virgin certificates, and hymen (re)constructions (The Swedish Government, 2025).

More than 20 years ago, the Swedish Association of Gynaecologists and Obstetrics (SFOG) recommended physicians to (re)construct the hymen only if a woman’s life were in danger. In 2022, SFOG released an opinion that supports the criminalisation of virginity control and operations without medical incentives as “surgical reconstruction of such a thin tissue as the hymen is difficult and can lead to changes in the surrounding tissues” (SFOG, 2022).

There are few empirical interview studies that have uncovered how young women perceive honor codes (either as social practices or ideological constructs) prescribe how both men and women should maintain the social reputation of their family (Christianson et al., 2021). Recently, Ahmad (2023) interviewed 14 younger and middle-age Dutch ethnic minority women, about women’s decision-making processes when they deviated from feminine honor codes. In Sweden, the virginity norm was in focus in an interview study with 15 teenagers, both young girls and boys with various ethnic backgrounds (Cinthio, 2015).

To the best of our knowledge, no qualitative studies have explored the perceptions of young women who may have been exposed to virginity testing and hymen (re)construction from a critical inquiry approach, to uncover inequities, marginalization, and social injustice in everyday life (Charmaz, 2017). From a gender perspective, the aim was to analyse how young women living in Sweden with experiences of patriarchal chastity norms construct and understand virginity and what conditions, actions, and consequences follow when virginity is prioritized by patriarchal communities. Specific research questions covered women’s subjective meanings, perceptions and experiences of virginity, virginity testing, and hymen (re)construction.

## Method

Grounded theory is an inductive method that discovers, creates, and verifies the phenomenon it studies, in this case virginity, through systematic data collection and analysis (Strauss & Corbin, 1990). The inductive nature of grounded theory embraces both openness and flexibility, which make the method appealing. In a previously published grounded theory study, we focused on honor and health (Christianson et al., 2021). Here, we focus on women’s subjective meanings and experiences of virginity, virginity testing, and hymen (re)construction. We use a constructive grounded theory approach described by (Charmaz 2006; 2017), where the informants and researchers co-construct knowledge. In line with Charmaz’s (2017) critical qualitative inquiry, we used concepts such as oppression, inequality, and injustice to uncover, oppose, and find a novel research direction.

### Participants

A purposive sampling strategy was performed. Eligibility for participation in the study required the informants to be over 18 years of age and live in Sweden in places where honor codes are significant. Inclusion criteria included a background from Middle East and other contexts where honor is important. Women under the age of 18 years old and women who did not speak English or Swedish were excluded. Recruitment occurred between 2012 and 2015. Women were asked to participate in the study through key persons at youth clinics, women’s organisations working against violence against women, and healthcare centres. Snowball sampling generated one informant. In total, 14 informants who lived in the capital of Sweden and in a university town in the north part in Sweden agreed to participate in the study.

### Measures and Procedure

Data collection was performed by researchers experienced in interview techniques and grounded theory analysis. Before the interview started, the informants were given the opportunity to ask questions about the project. All interviews were carried out in a respectful manner where the women were encouraged to share their experiences and views. Eleven informants were born in MENA countries, and three informants were born in Sweden. Eight informants migrated with their parents to Sweden in their early childhood and have been studying in Swedish schools. Two informants migrated to Sweden when they were adults, and one informant came to Sweden as a refugee when she was a teenager. The participants were between 21 and 38 years of age (mean age of 24). At the time for the interview, their living situations varied from sheltered accommodations to living in a single household or living with a boyfriend, a husband, or parents. Seven women reported that they were single, two women were married, two were cohabiting, three were divorced and two were living with their parents. As far as the interviews could tell, the women were identifying themselves as heterosexual. Six women had university degrees, six studied at universities, and two had graduated from high school. Three of the women had children.

The in-depth interviews lasted between 60 and 90 min and took place at a location that the interviewees chose. One interview was conducted via telephone. The interview guide included broad themes about the interviewee’s upbringing and living conditions and beliefs about equality, honor, virginity, norms and values about sexuality and relationships. Furthermore, we addressed open questions about the informants’ perceptions of virginity control, virgin certificates, and hymen (re)constructions and their views on those who control virginity and perform the operations. Clarifying and follow-up questions were asked during the interviews to obtain a rich, detailed narrative. The informants agreed to have the interviews recorded. The interviews were transcribed verbatim.

#### Researchers’ Positionality and Preunderstanding

We are white Western women, academics with doctoral degrees in medical science, educated in gender theory, and experienced in qualitative methods such as grounded theory. We have performed research about the hymen for over two decades.

Before the study began, the women received written information about the study and provided oral and written informed consent. They were informed that their participation was voluntary and that they had the freedom to withdraw their participation at any time without explanation. Steps to preserve confidentiality were taken, including removing names and other identifying information. The data have been stored in line with the policies at Umeå University, Sweden.

### Analysis

We performed a grounded theory analysis in line with Charmaz (2006, 2017), starting with an initial coding where we stayed close with the data. Each author read the interviews and did a first open coding individually. These codes were provisional, capturing and condensing meanings and actions, as Charmaz (2006) claims, “[to] capture the phenomenon and grab the reader” (p. 47). Next, we compared and discussed our individual coding and merged codes that overlapped. To reflect an insider perspective, we stayed close to the data, preserving the fluidity of the experiences that the informants described, keeping their perspectives in mind.

Next, we coded incident to incident and looked at how the informants understood the phenomena without judging their explicit actions. Seeing the world through their eyes and understanding the logic of their experience brought fresh insights (Charmaz, 2006). We reconstructed the preliminary codes into in-vivo codes with significant meanings and developed codes into subcategories that formed the informants’ experiences. According to Charmaz (2006, 2017), focused coding is the second major phase in coding, where codes are more selective and conceptual. Focused coding requires decisions about which initial codes make the most analytic sense with respect to categorizing the data completely. Through this procedure, events, interactions, and perspectives came into analytic view. By comparing data to data, we developed the focused coding per Strauss and Corbin’s (1998, p. 125) guidance, where axial coding answers questions such as “when,” “where,” “why,” “who,” “how,” and “with what consequences” relating the subcategories to overarching categories. To organize our data and create a story line, we used the conceptualization of conditions, actions/interactions, and consequences per Charmaz’s suggestion (2006, p. 42–71). During this process, we discovered the preliminary emerging categories and subcategories of each of the categories and the links between them, which reflected how we made sense of the data.

To tell a coherent story, in the theoretical coding, we searched for the core concept, forming the substantial model of (inter)action. Here, in this late stage of theoretical coding, we were inspired by Crenshaw’s (1989) intersectional theory. By focusing on black women’s experiences and how these intersect with “race,” subordination, and discrimination, Crenshaw critically examined the problems with sexism and patriarchy in jurisprudence. The inclusion of intersectionality creates a broader understanding of virginity and sexuality and adds to a more holistic representation of marginalised experiences and the forces that created those experiences (Abrams et al., 2020). We addressed power dimensions and structures that shape injustice, which according to Hankivsky (2012), is crucial. In the very last phase of identifying the emerging core category, we discovered how the epistemology of ignorance (Tuana, 2006) intersected with gendered power relations and social contexts (Hammarström et al., 2014).

## Results

The results are starting with why virginity is understood as a troublesome condition for women. This is followed by a discussion of how women’s sexuality is controlled both in cultural and medical contexts. Next, the consequences of the intrusions of medicine and the roles of physicians are discussed. Finally, on a structural level, we present the core category, which emerged from the other categories.

### Category: Unequal Sexual Conditions for Women Compared with Men

#### Women’s Subordination in a Man’s World

Most of the informants claimed that a woman’s life course is significantly influenced by the concept of virginity. That is, the gender norm system perceives virginity as essential to both women’s and men’s identities, which are formed by values and cultural traditions. In addition, the informants connected virginity to power, status, and means, where a woman’s intrinsic value is subordinated to a man’s value. Therefore, not preserving female virginity, perceived as an unwritten rule, is seen as socially devastating, resulting in ostracization and even physical harm. In many cultures, virginity is cherished from early childhood. The informants noted that historical and present social conditions have significant and negative influence on their lives:

And my mother said, “I don’t want to do like grandmother did, I don’t want to lock you in, but I want to show you that I am careful with you”. Mum said, “I don’t want you to destroy the precious[ness] that you have […]”. Then I thought, to be a virgin that is beautiful. (Interview 6; Lebanon)

Many informants saw their mother’s discipline as a means to protecting their honor, emphasising their ideals about the value of preserving virginity, maintaining the “good girl” ideology. Most of the young women were reminded that losing virginity was irreversible. Saving their virginity until their wedding night was often naturalized. Having boyfriends and one-night stands were forbidden. Many informants believed that their views about virginity and sexuality (i.e., abstaining from sexual intercourse until marriage) were coloured by the views found in their parents’ countries of origin. Most of the women did not care about the norms of virginity, but they experienced anxiety about how they would be judged if they did not live up to the norm.

#### Sex Before Marriage Is Stigmatizing

Most of the informants believed that cultural traditions contributed to a distorted view on unmarried women’s sexuality, as these traditions connected sexual intercourse with shame and filth. The interviews revealed psychological and emotional dimensions related to losing one’s virginity. Many mothers taught their daughters that having sex before marriage was ugly, bad, and wrong, a mindset that the informants carried with them into adulthood. That is, losing one’s virginity had to be done with the right man, at the right time, and at the right place. If these conditions were not met, women would be stigmatized, marked as “whores,” a designation that would have implications on their self-image and their sexuality. Infused with the assumptions that sex is unsuitable, forbidden, disgusting, and shameful may inhibit the ability to enjoy sex and cause stress in intimate relationship. One informant related how internalised emotions of shame and guilt influenced her sexuality negatively:

The things that harm many women are that if you lose your virginity without being married, that’s equivalent with [being a] “whore,” and the self-perception to internalise that you are a “whore,” dirty, then you cannot enjoy sex because you feel dirty. And you will feel that sex is something disgusting, forbidden, and not nice. [. . .] I felt guilt. I felt that I had done something wrong; I felt that I was a disgusting woman. So, there are many psychological consequences because of this idea that virginity exists, and one should preserve it. (Interview 9, Iraq)

#### Less Constrained Rules for Men

The informants claimed that the double standard was unfair. Men and women have different expectations: unlike men, women were required to abstain from sex before marriage. If women challenged the sexual norms, they risked being exposed to degrading invectives such as “whore”. However, if men engaged in sex before marriage, they were seen as masculine. The norms were perceived to be unequal as men were not pressured to abstain from pre-marital sex. Several informants noted that women and men should be treated equally. They pointed out the deep hypocrisy in many global contexts, where expectations of honor and respectability only concerned women. These viewpoints were seen as permeating all levels in societies, from individual to structural levels. For example, at universities in the Middle East, female students were expected to abstain from sexual relations until marriage. For one Somali informant, the influence of religion and her family’s background significantly influenced her mindset on virginity:

Yes, yeah for me it’s important, not because of culture but because of religion, so it is important to not have sex before marriage and that passes for both girls and boys. As soon as you are a Muslim, it’s important and I think [speaks louder]. Many other things that are not allowed within Islam such as smoking and drinking, that’s something you can take back, but virginity, that’s a border that I think one shouldn’t cross because you are a virgin only once. (Interview 5, Somalia)

Many of the informants believed that their mother raised boys and girls alike but that their father was less strict towards sons. This difference in parenting styles could be the result of generational socially transferred values (hegemonic masculinities) as the father is concerned with ensuring sons understand their role when they become head of their own family. That is, women and men have very different expectations when comes to sexuality:

It’s a bit hard to think about it [speaks louder]! My dad, for example, has had many girls before my mother [. . .]. Yes, he has had sex prior to her and obviously nothing wrong with that but for her [to have sex] was wrong! Wrong for me and wrong for all girls! (Interview 6, Lebanon)

Parents might be disappointed with their sons’ perceived immoral sexual behavior, but they would never end their contact with their sons. Boys were seen as behaving less shameful if they “grasped the opportunity” to have sex when they could; even sex outside marriage seemed to be tacitly accepted as their sexual behavior could be blamed on their immaturity. Regardless of their sexual orientation, men could do anything they want. Many informants described men as having the freedom to act the way they wanted. That is, they could “run around” and have fun until they got married. It was seen as “normal” for men to have sex with non-presumptive wives, divorced women, or prostitutes, because men do not have the capacity to surpass their sexual desires:

Boys are raised to take the chance if they can, and that’s ok and fine as long if they handle it properly; even sex outside marriage is ok. (Interview 5, Somalia)

In general, the informants found the double standard regarding sexual behavior unfair. Specifically, the women found the traditional view of women as merchandise where women’s sexuality is possessed by men as hypocritical. That is, women must be virgins when married, but men are allowed to have pre-marital sex. This double standard, the informants revelated, has deep roots in the belief parents must protect their daughters’ virginity. Moreover, in some of the informants’ families, it was the father’s and brother’s duty to protect the girls in the family by protecting their “virginity.”

#### Socialized Into But Questioning the Myths of the Virgin Membrane

From the informant’s point of view, virginity is seen as “preserving” their virgin membrane. Obviously, the informant’s upbringing and context (i.e., rural, urban, and country of origin) informed their ideas about the virgin membrane. Many women claimed that during their childhood they believed in the existence of a virgin membrane as their parents and relatives had strong beliefs about the existence and importance of the virgin membrane. Some informants’ mothers told them that women have a virgin membrane that breaks during their first sexual intercourse, which caused women to bleed:

My mother said that it [virgin membrane] existed, and the gynaecologist said that one bled when riding a horse and those things. Do you get me? I don’t remember exactly what she said and if it existed one hundred percent, but she said that people bleed [during their first experience with intercourse]. (Interview 5, Somalia)

Accordingly, the informants imagined a virgin membrane, depicted as a thin, sometimes invisible, barrier. One woman posited that the virgin membrane symbolised a woman’s worth or value, a prize that her husband can take. Many of the informants noted that the narrative about a virgin membrane rupturing during first sexual intercourse caused psychological, social, and physical inhibitions. Losing one’s virgin membrane was perceived to cause negative reactions linked to moral decadence. According to one informant, Iraqi women worried about losing their virgin membrane even if they had never had sex. Several women had been told that physical activity, tampon use, or accidents could rupture the virgin membrane and therefore some parents did not want their daughters to use of tampons or participate in physical activities:

Well, that was the thing with tampons. So if I used tampons, then I was widening my virgin membrane, and [a man] would notice and feel that. And if I don’t use tampons, it would not be wider. Then it’s like I know how it is to have sex when I have a tampon. It has to do with this; only married women use tampons. (Interview 3, Egypt)

I know that my dad didn’t want me to bike or ride horses because those things would break the virgin membrane. If you are doing tough sports, the virgin membrane can break. He didn’t say that directly, but I could read between the lines that it was like this. (Interview 14, Turkey)

Some informants were incorrectly told that gynaecological examinations could cause a rupture and sexual intercourse caused bleeding. Other informants said that they had never heard about a virgin membrane. The majority started to question the existence of a virgin membrane during high school or senior high school. Menstruation made them suspicious that they had been fooled by their parents. Friends also mentioned that it was impossible to detect if women have had sex. Several of the informants were convinced that losing a virgin membrane during sexual intercourse was a myth. They believed that they had been brainwashed during their upbringing. They were disappointed. One informant stressed that in the Quran nothing was written about a virgin membrane, and she explained the root cause of bleeding:

So, for us**,** they have put stiches in the opening and if it has been opened, one will see that one has done something. And that is why we are bleeding as it is difficult to have sex as the opening is so little. Rifts and ruptures occur and that’s why we are bleeding, and it is not that we are losing our virginity and things like that. (Interview 12, Somali)

Another woman stressed that physical activities may affect the membrane:

I knew that the virgin membrane is a myth. I have read that it wasn’t a membrane but rather skin, and I was dancing a lot, and I have read that it can disappear when one is doing special moves. (Interview 13, Iraq)

One informant thought that it was unwise to believe in a virgin membrane as she did not find evidence of its existence:

I know that there exists a myth about that it [the virgin membrane] ruptures during the first sexual intercourse, and one bleeds a little bit, but there are women who are not born with it, and there are women where it does not rupture until they give birth. It differs. It’s nothing you can type [as] a sign that you are a virgin, it’s a sign that this is the first man. It’s stupid to believe such things. Basic grounds [for such reasoning] are missing. (Interview 2, Lebanon)

The informants believed that all the talk about virgin membranes is a type of gender inequality designed to control women and repress their sexual autonomy.

### Category: The Making and Faking of a Virgin

#### The Need to Control Women’s Virginity

Several cultural narratives explained how and why women’s virginity should be controlled. One informant was told by men that they could tell whether women were virgins by looking to see whether women had a “more open corner of the eye” or “more glossy eyes” or by how a woman moved her body. In addition, some informants were told that a female bikini waxer could tell whether a customer was a virgin. Sometimes older women were seen as oppressors by the upholding the virginity standard. One informant had been told by her mother how young women sometimes were brought to a woman who examined the state of virginity by putting one or several fingers in the opening of the vagina. Apart from verifying virginity, some women could discover how much sex a woman has had. For example, a religious woman (a wife of a priest) “ran a business for men” by checking virginity:

And the woman is laying down and spreading her legs. And then this person, the wife of a priest, she looks down, and I don’t know if she puts her fingers or if she just takes a look. This looks good this doesn’t look good, and the horrible thing is that when she leaves the room, then she, the wife of the priest, does this happy shout [to celebrate that the woman is a virgin]. (Interview 4, Iraq)

#### The Blood-Stained Sheet

Some informants had heard stories about how blood-stained sheets are used to confirm a wife’s pre-marital virginity. One informant claimed that virginity certificates were not necessary if a blood-stained sheet was displayed after a wedding. Another informant mentioned that in Lebanon this was a common practice in the 1980s, and her sister was required to show the bloody sheet to confirm she was a virgin when she married. The husband or another woman verified that bleeding had occurred, and the bloody sheet was presented to the wife’s mother-in-law. Often, the whole family gathered outside the house after a marriage was consummated to view the blood-stained sheet. When the blood sheet was displayed, the audience would shout with joy:

And when my brother married, the bloody sheets were shown per the tradition. Sometimes many relatives stand outside to see [the evidence of virginity]. It was the same thing when my second brother married, and I said to my mother, What is this? According to the tradition, my mother and the mother-in-law confirmed the virginity, but my mother said, “I don’t like it. I don’t care [for the tradition].” (Interview 10, Lebanon)

The informants mentioned that wedding night was characterised by anxiety, stress, and pain. Many of the women (and men) were nervous that the evidence would not be convincing as a lack of blood would be a catastrophe. In addition, some men spread rumors that the wife “did not bleed on the wedding night”. Many of the informants noted that showing a blood-stained sheet was an infringement on women’s dignity and rights. Moreover, the informants believed that it was demeaning when parents demanded a virginity test. Some informants knew that young women are worried that they would not bleed on their weeding night. One informant told a story about a friend who cut herself to ensure she would pass the blood-stained sheet test. Many informants understood that not all women bleed after their first sexual intercourse, so it is impossible to know whether a woman is a virgin using the blood-stained sheet test.

#### Verifying a Virgin via the Virgin Certificate

The informants pointed out that a woman’s virginity is controlled independent of class position. Few women had personal experiences of virgin certificate, but they claimed that the phenomenon occurred globally. Several informants believed that in the Middle East, especially in Iran and Iraqi, a virginity certificate is required before a woman can marry. They believed a gynaecologist could verify whether a woman is a virgin. For example, in Iranian women often sees a doctor to obtain a virgin certificate. Obtaining a virgin certificate was perceived as very important for the family of the woman and was mentioned as a ticket to marriage. After a divorce, a virgin certificate was perceived as to be helpful if the woman wanted to remarry.

The few informants who had experience with virgin certificates talked about the pressure from their families to undergo an examination to receive a virgin certificate. In one case, the mother changed her mind when the daughter approved to be examined. In another case, a mother arranged the appointment with a doctor in Iran, because the mother was suspicious that her daughter had had sex. Her mother followed her to the examination, and the informant remembered how embarrassing it was to expose herself in front of the doctor:

It’s totally normal and my mother said okay we must see a doctor to get the paper [virgin certificate]. You are going to get married, and all women do this before marriage. And I was so ashamed. I was so nervous. I was ashamed, but I said okay I must go there. And I went. And when the doctor wanted to examine me, I was so ashamed. But it’s better to get this paper so the groom can see that you were a virgin. (Interview 8, Iran)

The doctor confirmed that everything looked normal–i.e., she was “a proper young woman”–and signed the two copies of the certificate, one for her mother and one for the potential husband and his family. According to the informant, a virgin certificate could minimise the risk for being exposed to shame. Contradictory views were also seen. The informants’ reflections supported the idea that medical ignorance and traditions were the basis for virginity testing, a form of control over women. One informant talked about her mother who was forced to marry at a young age. She emphasised that gender and power were intertwined with traditions and harmful values, resulting in humiliating treatment that restricted young women’s opportunities. Virgin certificates were seen as disruptive, an abusive practice with many consequences for women’s mental and sexual health, including risk for self-harm and involvement in unhealthy sexual relations.

### Category: Surgical Interventions in Gendered Bodies

None of the women had experience with hymen (re)construction, but many had heard about the operation. They did not understand how the operation was done as they have heard that the virgin membrane was fragile. They believed that hymen (re)constructions were done in secrecy. Some informants were unsure if hymen (re)constructions were performed in Sweden, and some assumed that it was forbidden in Sweden:

Sad that women chose a hymen reconstruction. Women are exposed, and I am not sure that this is done in Sweden, but I know that they do it in Denmark. (Interview 4, Iraq)

Some informants mentioned that the operation occurred in the Middle East and that women go to hospitals to get the stiches. It was claimed that educated people at the universities believe that the virgin membrane could be verified and reconstructed. They had vivid opinions about the phenomenon. Rumors circulated that women could restore their virgin membrane before marriage. One informant believed that young women who had had sexual relations before marriage could be “fixed up” via an operation or with a less invasive method:

I was not a virgin when I married a Turk. I was scared to death. I went to the youth clinic and talked to the woman I always talked to when I needed oral contraceptives and things like that, and she started to talk about operations and took it up, those possibilities, but it wasn’t something they recommended [. . .] was it so important [. . .]? But in that panic, it was really important, [as] I was going to marry in Turkey and one had heard horror stories about people who did not bleed on the wedding night and had been killed with a gun […] I solved it [the problem] by stop taking the pills and then I got my bleeding [by timing my bleeding] with my wedding night. (Interview 1, Iraq)

One informant said her mother worried that her tampon use would destroy the virgin membrane, so the mother suggested an operation to safeguard that her virginity remained intact. She calmed her mother and refused to do this, as she believed this was an unnecessary operation.

#### Hymen (re)Construction Is Wrong

Hymen (re)construction was viewed as a very complex matter and raised negative emotions among the informants. One informant thought that it was sad that hymen (re)constructions are performed and that they are only advocated for by unwise and uneducated people. Some informants pointed out that the real problem belonged to the family, husband, or society, not an individual woman, but the consequences for women were seen as very harsh. Therefore, women sometimes were forced to have an operation. If requirements of virginity did not exist and if relatives and cultures did not demand blood-stained sheets on the wedding night, hymen (re)constructions would not happen. Many claimed that oppression of women was the fundamental motivation for hymen (re)construction, as women seldom choose a hymen (re)construction for themselves. It was seen as sad and upsetting that women were restricted and could not make autonomous decisions. It was seen as a crime against women’s human rights. One woman thought that physicians were acting unethically:

I think that it [hymen (re)construction] is wrong. Consequently, I don’t think that it should exist. So, on what grounds are doctors doing it? Sure, you believe, or you don’t believe. I guess that it’s only done for the money, as it’s the money they want. But you know, at the heart this is about oppression: is it worth the money? So, I cannot understand. I think that it’s so wrong. So, I guess that one does it in pure desperation for sure. But I cannot understand that a doctor says yes to this; I don’t think it is ethical. Sure enough, I think this is as bad as FGM [female genital mutilation]. (Interview 13, Iraq)

Hymen (re)construction was viewed as tragic, terrible, and wrong and sometimes compared to FGM and therefore the operation should be forbidden. The view was that the operation was a form of mental and physical abuse, violating the woman’s sense of self agency. The informants claimed that if women did not resist hymen (re)construction, they implicitly accept that others would make decisions for them. In a fair world, hymen (re)constructions would not take place. Many of the informants believed that hymen (re)construction was seen as unacceptable in Sweden.

#### Criticizing Doctors who Perform Fake Operations

The informants criticized doctors who spread false information about the existence of a virgin membrane. “On television in Turkey, doctors inform the audience, especially housewives, about virginity and virgin membranes.” Some informants believed that doctors have a responsibility to think about what is best for the woman rather than what would satisfy cultural and religious expectations. One informant challenged the rationale for the operations, as she believed the practice is based on myths and ancient cultural norms about a virgin membrane:

But at the same time, when doctors accept to do those operations what signals are they sending out? Because in the Middle East they do these operations and they make this myth to continue to live, that a girl can have the stiches, she bleeds, and she has a membrane that will rupture. All those things. (Interview 7, Iran)

When performing the operations, doctors place women in a vulnerable situation as it forces bleeding on the wedding night. They thought that doctors were simply naïve if they believed that women requested an operation free of cultural, religious, and familial pressures. The informants thought that doctors are aware that women are oppressed, but they still perform the operations, a practice that was seen as unethical, demonstrating a lack of respect for women. For the informants, doctors seemed to be encouraging other health personnel to keep up the practice. However, the informants wanted doctors to act ethically: to advise their patients to not partake in the practice even though it meant that they would make less money. The informants thought that making money was the main reason doctors recommended hymen (re)construction. However, hymen (re)construction was also seen as a Catch-22, both as unethical and a life-saving intervention (i.e., an intervention that could save a woman from honor killing).

#### Understanding Hymen (re)Constructions as the Last Solution

The informants emphasised that condemning doctors who were willing to do these fake operations was not fruitful as long as doctors are unwilling to contest the existence of a virgin membrane:

And it doesn’t seem to be commonly known that the virgin membrane doesn’t exist. Or like this. I have a friend who is a doctor, and I talked to him about it, and I said, a virgin membrane does not exist, and he just said, yeah, the virgin membrane does exist. And I just . . . nope. Because of that [this belief], it feels like the take-off-point is that everyone believes that it exists, so I think that it is very good that a girl has this alternative, a life-saving type of operation. When it is a matter of life and death, then I think it is good that there are doctors who are willing to perform such an operation. (Interview 11, Iraq)

Many young women worry that they will not bleed on her wedding night, so they seek the operation. Some informants saw that it was a good situation that they could have pre-marital sex and then have an operation to “correct their former mistake” in secrecy. This was seen as a short cut if proof of virginity was demanded:

These are good thoughts. Because women who are enjoying sex and have done what they want, and later when they are going to marry, they can do this shit operation because the family will not accept them otherwise. (Interview 12, Somali)

Young women who had not had sex could also ask for the operation since not all young women bleed during first vaginal intercourse. Parents or relatives were perceived as continuing to be unknowledgeable about the fact that young women nowadays are breaking norms by having pre-marital sex. Sometimes parents also suggest an operation before marriage as a rumor-preventing step. Most informants claimed that it would be unethical to refrain from providing this opportunity to young women in danger. Some women found that the operation could be an advantage such as calming a young woman who had reasons to be scared. From the informants’ point of view, if a woman seeks the operation, she has motives as all threats against a young woman must be taken seriously:

In the worst case she will be murdered if she is not a virgin…it is very, very, very difficult. At the heart of the situation, I don’t think one should do the op…but for me, it would be very difficult if I were the physician and met an immigrant-dolly who risked her life because she is not a virgin…to refrain from helping her. But it feels wrong…very frustrating […] letting this cultural mindset still exist when one does those operations. (Interview 3, Lebanon)

Hymen (re)construction was not regarded as acceptable but was perceived as a strategy to avoid the shame of not bleeding on the wedding night or a strategy to escape violence or the risk of abandonment. Being able to confirm one’s premarital virginity was seen as a matter of life and death. Hymen (re)constructions were talked about as life-saving procedures, as they could prevent a woman from being murdered or committing suicide. The informants concluded that many young women from cultures outside Sweden have offered their lives for freedom, and they claimed that in Sweden the operations cannot be banned. Some of them compared hymen (re)constructions with cosmetic surgery and thought that 20 000 SEK was a cheap cost for staying alive.

### Core Category: Intersecting Dimensions of Honor Cultures, (un)Medical Power and Gender Injustice Sustain the Norms of Virginity

Tuana’s (2006) taxonomy of ignorance towards women’s bodies and reproductive phenomena provides a valuable frame for understanding how the epistemologies of ignorance are connected to the concept of virginity and the hymen. The impact of ignorance in various cultural contexts resembles how the power of medical authorities and their inconsistencies in conveying anatomical knowledge perpetuate gender injustice. A veil of ignorance implies that both grass root people and medical authorities often rely more on myths than anatomical facts when it comes to women’s genital anatomy. In short, cultural needs and patriarchal structures, not anatomical facts, have driven the morphology of virginity and the hymen, and the practices of virginity testing and hymen (re)constructions.

## Discussion

**Fig. 1:**
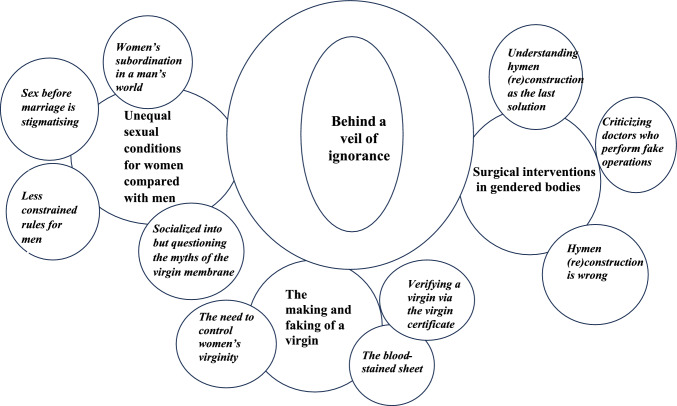
A flow chart of how the perceptions of virginity and hymen (re)constructions permeate through women’s life spaces and intersect with a veil of ignorance and a web of gender injustice

To embrace the multidimensionality of experiences that our data represent, the emergent conceptual model synthesises how honor cultures interact with (un)medical power and gender injustice, sustaining norms of virginity by controlling women’s sexuality (Fig. 1). In our interpretation, virginity testing, and hymen (re)construction are part of a complex web of gender inequality. Our analysis reveals how the informants’ constructive narratives of sexuality and morality are collectively influenced by both time and place. These structures change over time as human practices change. Most informants were studying or had university degrees at the time of the interviews and their backgrounds may have contributed to their reflections on gender injustice.

Using a multilevel approach in our model, we address how the concept of virginity contributes to disempowering women in contemporary societies on several levels: the individual—i.e., the woman’s personal experiences; the institutional—i.e., how the family traditions and the medical authorities collectively influence and restrict the (bodily) autonomy of women; the structural—i.e., how the gender order shapes gender oppression; and the historical—i.e., how the informant’s background and historical context may contribute to a broader picture of the underlying dilemmas with the norms of virginity in contemporary societies (The Center for Intersectional Justice, 2019).

Although virginity testing and hymen (re)construction are old practices (Sissa, 2013), they are still used within medical practices globally (Saharso, 2022; WHO, 2018; Wisniewska-Slepaczuk et al., 2022). Here, we investigate why these out-dated and medically questionable practises are still being used. Some forensic experts are very critical of virginity testing and want to ban it (Zayed et al., 2022). Gynaecologists and general practitioners in Sweden seem to be unwilling to perform virginity certificates or hymen (re)construction (Juth & Lyneö, 2015). Hence, virginity certificates and hymen (re)constructions concern women much more than men as women’s experiences are grounded in living in a gendered world where unequal conditions and the ideology of virginity impact women’s health more than men’s health.

The category unequal sexual conditions for women compared with men underscores how many of the informants perceived that they had been indoctrinated about the importance of abstaining from sex to become honorable and marriageable, which we interpret as oppressive social/sexual norms women must bear in childhood as well as adulthood. The data reveal a clash between tradition and modernity, as many of the informants questioned whether the virgin membrane exists, as information that the virgin membrane is a myth has reached the general public in Sweden (Christianson & Eriksson, 2004,2011; The Swedish Association for Sexuality Education (RFSU) 2009, UMO, nd), and in groups where the cultural understanding of honor is an important value (Cinthio, 2015). Knowing facts about the hymen does not solve the dilemmas for young women. That is, their narratives suggest that mothers, relatives, men, and other groups in the society, including medical personnel, unconsciously contribute to upholding this veil of ignorance (Tuana, 2006).

Virginity is placed at the intersection of medical (un)knowledge, relationship rules, and moral values. In the category The faking and making of a virgin the informants’ narratives reveal past and present symbolic stories of breakable hymens and how blood is intrinsically linked to virginity. Few of the informants talked about their own experience of bleeding but anecdotally elicited various events where the blood-stained sheet was seen as central in some cultures. The role of healthcare professionals (mainly doctors) is controversial as they may be accused of upholding the myths of virginity by doing virginity tests and thereby, according to Crosby et al. (2020), act as “moral police”. A virginity test has no benefits for the patient/woman as it is an unnecessary practice that has emotional and physical health risks. The harmful side effects of the practices (e.g., discomfort, pain, and depression) overshadow any positive effects for women.

Many researchers and stakeholders believe that virginity tests are unscientific and violate the right to avoid unnecessary examinations, as in most cases a virginity examination is demanded by people other than the girl/woman herself (Behrens, 2015; Koth & Abo-Zeid, 2022; WHO, 2018). According to Moaddab et al. (2016), from an ethical point of view, a virginity test is not compatible with professional accountability in medical science, as having sex is not a pathological condition that requires medical care. Moaddad et al. (2016) discuss virginity testing framed by ethical codes of conduct among physicians—i.e., medical decisions should be based in beneficence, autonomy, and justice and all healthcare providers should protect and promote the biopsychosocial health of women. They argue that virginity testing violates the mandate that healthcare providers should protect women’s human rights, and that legalisation should be created that makes virginity testing illegal. Crosby et al. (2020) point at the complexities surrounding virginity testing and the challenges to change social norms such as marriageability and social status for women. They recommend a softer approach that relies on education and the spread of information about the harms associated with virginity testing while also advocating for global elimination over time.

A mixed method study from USA about beliefs on virginity among healthcare providers and medical students indicates that participants have vague ideas about anatomy (Walsher et al., 2023). In their study, a majority responded that girls are born with a membrane, a view that is in line with a global web survey among midwives concerning their beliefs about virginity and the hymen (Christianson & Eriksson, 2013). This lack of or ambivalent knowledge about virginity and the hymen is precisely in line with Tuana’s (2006) elaboration in her essay “The Speculum of Ignorance”, where she suggests that if knowledge is not valuable for privileged groups like medical personnel, they are “not caring to know” (p. 5). From our point of view, education in medicine is recommended to be revised and updated so that misinformation about women’s genital anatomy and the mythology of the hymen end. Although virginity is not a biological entity, the belief that virginity testing works in many settings as a control of sexuality harms women’s social, physical, psychological, sexual, and reproductive health (Crosby et al., 2020; Militz, 2022; WHO, 2018; Wild et al., 2015). According to a 2018 WHO statement directed to governments, healthcare providers as well as national and regional health agencies should eradicate all forms of virginity testing (p. 16).

However, the progress of ending virginity testing is slow. Many informants also pointed at the pressure of wedding night traditions in some of their cultures, which we believe could be linked to sexual violence. Discussions and definitions of honor-based violence tend to focus primarily on direct forms of violence. However, less attention has been given to the complex use of violence, which is often more subtle and indirect, such as silencing and threats to enforce conformity to honor codes (Ahmad, 2023; Christianson et al., 2021). Thus, in addressing violence against women in honor-related contexts, it is crucial to also include and acknowledge the adverse health consequences resulting from the more intangible aspects of coercive control over women’s bodily autonomy.

Very few of the informants conceptually recognised virginity tests or hymen (re)constructions as sexual abuse. This view may be influenced by the desire not to question medical authorities, including how they perform virginity testing, which could be seen as sexual violence. According to Cohen Shabot (2020), this type of sexual violence is obstructed and perfectly matched with the “normal” embodied situations for women living in patriarchy and therefore the doctors and the women do not see the actions as violence, especially in a medical setting, where the rules of professional ethics are assumed to be enforced.

Medical providers, especially physicians, are often highly respected. Interestingly, in the category Surgical interventions in gendered bodies, the informants raised both critiques and understanding towards the physicians who perform hymen (re)constructions. On the one hand, the informants emphasised that, although hymen (re)constructions are unethical, it may be a lifesaver for women. On the other hand, some informants believed that medical personnel who perform hymen (re)constructions are merely upholding wrong-headed ancient traditions and consequently reinforcing unscientific practices and perpetuating gender and sexual inequalities. Pace et al. (2023) found that societies and communities often pressure women at risk for honor violence to seek hymen (re)construction, a situation that replaces one risk for another risk rather than solving the problem – i.e., relieving vulnerable women of bodily and psychological harm altogether. The informants in our study were suspicious of the motives behind the operations by claiming that physicians may see hymen (re)construction as way to make more money (cf. Wisniewska-Slepaczuk et al., 2022). In an interview study from Tunisia, physicians admitted that financial gain motivated their use of hymen (re)constructions (Wild et al., 2015). On one French homepage, the price for a hymen (re)construction is 3000 euros, and on a Swedish homepage in 2024, the cost is approximately 15 000–18 000 Swedish crowns. Tuana (2006) believes that the veil of ignorance is in part due to physicians having “no interest in coming to know” the facts about women’s health (p. 6).

Our findings point to how norms of virginity lead to gender inequality and negative effects on women’s sexual and reproductive health. In an earlier published paper, we discussed the need to raise awareness and educate people about the myths of the hymen and promote women’s human rights (Christianson & Eriksson, 2015). Many other studies have raised similar concerns (Crosby et al., 2020; Moaddad et al., 2016; Pace et al., 2023; Wild et al., 2015) and call for proper counselling for women about genital anatomy and human rights. In Sweden, by 2025 both virginity testing and hymen(re)constructions will be considered illegal (Government Offices of Sweden, 2022).

Although gender research voicing the viewpoints of racialised minority groups in Sweden are rare, there is an ongoing broader debate concerning the polemics of viewing virginity control and hymen (re)constructions as pragmatic solutions for migrant and other women (Saharso, 2022) or as a violation of women’s human rights (WHO, 2018). A benchmark is to judge migrant women as less autonomous and in need of anatomical knowledge about their genitals compared to perceived informed white western women. During women’s health movements in the USA between 1960 and 1970, an epistemic practice to undermine ignorance was the genital self-exam (Tuana, 2006), which is a practice without side effects that still can be used today.

### Methodological Strenghs and Limitations

According to Birks and Mills (2015), there are some distinct features of how to achieve quality and rigor in a grounded theory study, including the researchers’ expertise and skills, how well the aim and method harmonise, and the precision of procedures. Here, we apply a constructivist grounded theory in line with Charmaz (2006, 2017) as such a methodology is congruent with our research question. That is, informants helped us construct the meanings of virginity, an approach that is a strength of this study. The analytical conceptualisation combined with the use of gender and intersectionality added depth to the emerging core category, which is revealed in our analysis. We have been transparent with our philosophical gender perspective position, also a strength of this study. A limitation may have been that the informants’ various backgrounds included a risk for under-reporting. Another drawback may be that the sample size is rather small. However, according to a recent publication by Dworkin (2024), also small-scale studies with 10–15 informants can generate robust data if saturation has been reached. In grounded theory, the concept theoretical saturation is used and according to Charmaz (2006), the theoretical saturation is reached when the categories are fully developed, and further analysis adds little to the result. We carefully explained the process of the analysis to make the result trustworthy. To stimulate theoretical sensitivity, the use of intersectionality and philosophical literature (Crenshaw, 1989; Tuana, 2006) provided ways to approach and interpret the data which is a strategy to increase validation.

### Conclusion

The core category—Intersecting dimensions of honor cultures, (un)medical power, and gender injustice sustain the myths of virginity—reflects how history, cultures, families, medicine, and societies influence how the interviewed young women understand how the unequal gender order manifests the norms of virginity and why they are both critical and sympathetic of those who perform virginity control and hymen (re)constructions. Our analysis reveals that patriarchy, a system of social structures and practices in which women are oppressed and exploited (Walby, 1990), affects young women’s well-being and health. However, dismantling patriarchy is possible by raising awareness of reproductive inequalities and reducing the “epistemologies of ignorance” (Tuana, 2006) that surround the myths about the hymen. To lift the veil of ignorance, we raise awareness among laypeople, medical professionals, and students about the unscientific assumptions underlying the concept of virginity. Legislation, although disputable, may facilitate the ending of long-lasting myths about a breakable membrane, but first medical personnel must acknowledge that virginity is a social construct. Future research should focus on young men and their perceptions on virginity, virginity control and hymen (re)constructions.

## Data Availability

Not applicable.
